# Microwave-Assisted Synthesis of (C_12_H_25_NH_3_)_2_MCl_4_ Hybrid Perovskites (M: Zn, Cu, and Mn): Metal-Ion Effects on Structural and Thermal Properties

**DOI:** 10.1021/acsomega.6c00060

**Published:** 2026-07-26

**Authors:** Ali Mirahmad, Ravi Shankar Kumar, Breogán Pato-Doldán, Javier Díez-Sierra

**Affiliations:** † Thermal Energy Storage Department, Iberian Center for Research in Energy Storage (CIIAE), 10004 Cáceres, Spain; ‡ Department of Energy Engineering, Universidad de Sevilla, 41092 Sevilla, Spain

## Abstract

Hybrid perovskites are emerging as promising candidates for thermal management and thermal energy storage (TES). Compared to traditional solid–liquid phase change materials, these hybrids offer tunable structures, enhanced thermal conductivity, and comparable latent heats. This study extends a rapid, scalable microwave (MW)-assisted synthesis to a broader suite of organic–inorganic hybrid perovskites with the general formula (C_12_H_25_NH_3_)_2_MCl_4_ (where M = Zn, Cu, Mn). Beyond broadening the synthetic scope, we provide a comprehensive comparative analysis of how metal center substitutions influence material properties. The structural integrity, morphology, and phase purity were systematically characterized via XRD, SEM, EDX, and FTIR, while thermal behaviors were evaluated using DSC and TGA. The results demonstrate that the specific metal ion significantly dictates both the crystalline structure and phase transition thermodynamics. Transition enthalpies ranged from 58 to 112 J/g, with peak temperatures between 46 and 74 °C. Thermal stability also varied across the series, with decomposition onset occurring between 270 and 335 °C. By elucidating how metal selection steers performance, this work establishes MW-assisted synthesis as a versatile, energy-efficient platform for the tailored fabrication of advanced solid–solid phase change materials for practical energy storage applications.

## Graphical abstract

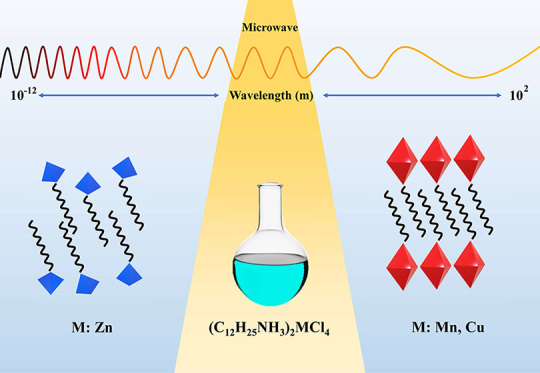

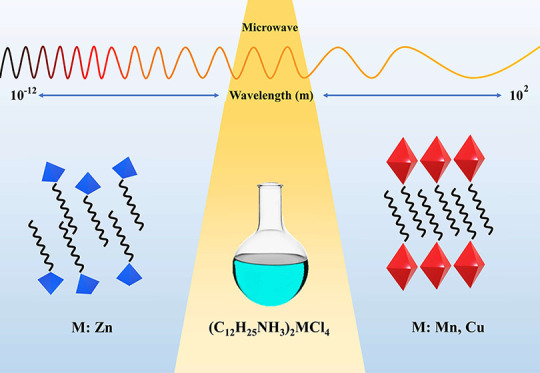

## 1 Introduction

Phase Change Materials (PCMs) have gained considerable attention in recent decades as promising candidates for efficient thermal energy storage (TES) applications.^1,2^ These materials store and release thermal energy through phase transitions, typically solid–solid, solid–liquid, or liquid–gas.^3–5^ PCMs have been extensively explored for applications such as solar energy storage,^6,7^ waste heat recovery,^8,9^ building temperature regulation,^10,11^ and thermal management in electronic devices.^12,13^ However, many solid–liquid PCMs suffer from leakage and noticeable volume change during melting.^14,15^ To overcome these issues, solid–solid PCMs become an alternative solution that stores latent heat through reversible structural changes while remaining in the solid state.^16,17^ This prevents leakage and reduces volume change, making them suitable for various thermal management applications. The solid–solid PCMs particularly became attractive for applications that require structural integrity and minimal maintenance compared to solid–liquid PCMs. Among the various solid–solid PCMs, hybrid organic–inorganic perovskites have recently attracted increasing interest due to their unique ability to undergo tunable and reversible solid–solid phase transitions, which can be precisely controlled by changing the organic cation or metal ion in the structure, offering flexibility for diverse thermal energy storage applications. They also exhibit relatively high enthalpies for these transitions, combined with good thermal stability and low thermal hysteresis, making them particularly promising candidates for efficient and sustainable thermal energy storage.^5,18–20^ The organic–inorganic halide perovskites bis­(alkylammonium) tetrahalometallates (II), generally denoted as (C_n_H_2n+1_NH_3_)_2_MX_4_, where C_n_H_2n+1_NH_3_ represents a long-chain alkylammonium (n = 1 to 18), M represents a transition metal, and X is a halide ion, normally possess a typical two-dimensional layered structure.^21–28^ The stacked organic cation layers and inorganic metal-halide octahedral^5^ (in some cases tetrahedral^19^) layers allow precise tuning of their thermal and physical properties by varying the organic chain length, halide type, or transition metal ions.^20^ In these hybrid materials, the absence of strong covalent bonding between the organic and inorganic components results in enhanced structural flexibility.^5^ Above room temperature, two distinct phase transitions are observed: a low-energy reorientation of the ammonium groups and a higher–energy transition characterized by increased chain disorder and interlayer expansion.^5^ These structural changes are critical as they can significantly influence properties such as exciton dynamics, which are essential for applications in optoelectronic devices.

The phase transitions in hybrid perovskites are governed by the motion of the organic component, while the inorganic octahedral framework largely remains rigid or shows planar movement, consistent with the crystalline bilayer model.^29–31^ For example, Cascos et al.^30^ showed that changes in the ordering of organic cations can modify octahedral tilting in the inorganic layers, leading to symmetry shifts during thermal phase transitions. Raj et al.^28^ further confirmed that these transitions involve changes in lattice symmetry and structural ordering, as evidenced by peak shifts in XRD patterns for manganese-based hybrid perovskites (1-C_n_H_2n+1_NH_3_)_2_MnCl_4_ (n = 9 and 10), prepared using a solvothermal reflux method. They reported the first phase change occurring between 17–21 °C with a latent heat of approximately 64.75 J/g for n = 9, and between 35–38 °C with a latent heat of approximately 73.83 J/g for n = 10.^28^

These compounds are usually produced by reacting stoichiometric amounts of an alkylamine (the A-site precursor) with a divalent metal halide (the M-site precursor) in the presence of a surplus of the relevant hydrohalic acid (HX). The reaction is performed under continuous reflux in an appropriate solvent for a period of 2 to 5 h. However, this method often faces challenges such as prolonged reaction times, uneven heating, and higher energy demands for synthesis.^5^ (C_12_H_25_NH_3_)_2_ZnCl_4_ was synthesized using the solvothermal method by Abdel-Kader et al.^32^ The phase change observed at 89.9 °C showed a phase change enthalpy of approximately 133 JK^–1^ mol^–1^. Raj et al.^28^ synthesized manganese-based halide perovskite particles with an average size of 20 μm, demonstrating their robust stability over 1000 continuous heating and cooling cycles. In another study, Salgado-Pizarro et al.^27^ studied the optimization of the solvothermal synthesis method for producing (C_12_H_25_NH_3_)_2_CuCl_4_, comparing processes with and without recrystallization. The findings revealed that isopropanol was the most effective solvent, achieving the highest phase transition enthalpy. Although combining solvothermal synthesis with recrystallization resulted in the greatest crystallinity, it caused considerable material loss during filtration. Therefore, the method without recrystallization was deemed the most efficient for large-scale production. Furthermore, solvent-free reactions have also been utilized to synthesize halide perovskites using ball milling.^26,33,34^ Salgado et al.^26^ successfully produced bis­(dodecylammonium) tetrachlorocuprate through a 12 min ball milling process, achieving results comparable to those obtained by conventional solvent-based synthesis. However, this approach presents certain limitations, including post-treatment time,^26^ sensitivity to reagent particle size,^34^ a higher risk of contamination,^35^ reduced crystallinity,^36^ and difficulties in reproducibility and scalability of the process.^37^

To address these limitations, the MW-assisted synthesis method has emerged as an energy-efficient and scalable alternative. This method offers faster reaction kinetics (shorter reaction times) and more uniform heating compared to the conventional approach.^38,39^ The underlying mechanism of MW-assisted synthesis is the direct interaction of the oscillating microwave field with polar molecules and ionic species in the precursor mixture, which generates rapid volumetric heating via dipolar polarization and ionic conduction.^40,41^ This uniform heating facilitates nucleation throughout the reaction medium, enabling the formation of highly crystalline materials.^5^ In our previous work,^5^ we introduced a MW synthesis approach for (C_12_H_25_NH_3_)_2_MnCl_4_. This method not only significantly reduced the required reaction time but also yielded materials with superior properties, including enhanced crystallinity, higher phase transition enthalpies, and improved thermal stability. To further validate the effectiveness of the microwave technique, the present work expands this methodology to other members of the hybrid perovskite family through a comparative analysis. This comparative investigation evaluates the thermophysical properties of various derivatives, (C_12_H_25_NH_3_)_2_MCl_4_ (where M = Mn, Cu, and Zn), specifically highlighting how the choice of the transition metal influences the final performance of the synthesized materials. Our findings demonstrate that MW-assisted synthesis is a versatile and efficient general approach for producing high-quality hybrid perovskites, particularly for applications in thermal energy storage.^5^ Among the synthesized sample, (C_12_H_25_NH_3_)_2_ZnCl_4_, exhibited the highest phase transition enthalpy at the highest temperature.

## 2 Experimental Work

### 2.1 Materials

1-Dodecylamine hydrochloride (99% purity), Magnesium chloride (MnCl_2_) anhydrous (99% purity), Zinc chloride (ZnCl_2_) anhydrous (99% purity), and Copper­(II) chloride monohydrate (CuCl_2_·H_2_O; 99% purity), were used as received from Sigma-Aldrich. Hydrochloric acid (HCl, concentration 37%) and Isopropanol (≥99%) were purchased from Thermo Scientific and used without any modification. Before the experiments, all the glassware was prudently cleaned with laboratory-grade ethanol, followed by hot oven drying for 30 min at 100 °C to ensure the removal of any chemical contaminants.

### 2.2 Synthesis Procedure Using Microwave Route

The hybrid perovskite compounds were prepared by reacting 1-dodecylamine hydrochloride with a metal salt in a 2:1 molar ratio, following the reaction described in Table 1. The synthesis of hybrid perovskites using the MW route was carried out in two steps. 1-Dodecylamine hydrochloride (1.553 g, 7.00 mmol) was dissolved in 15.0 mL of isopropanol to prepare Solution A. Separately, the metal chloride salts (3.50 mmol) of ZnCl_2_ (0.477 g), CuCl_2_·2H_2_O (0.597 g), and MnCl_2_ (0.440 g) were dissolved in 10.0 mL of isopropanol to prepare Solution B. Both solutions were combined and stirred at 400 rpm for 30 min at room temperature, as shown in Figure 1. Then, ∼0.5 mL of HCl was added dropwise to each solution and stirred for 5 min (Solution C). Solution C with a final volume of ∼25.0 mL, giving final concentrations of amine = 0.280 M and metal = 0.140 M, maintaining the 2:1 stoichiometric ratio required for the formation of (C_12_H_25_NH_3_)_2_MCl_4_. In the second step, the final mixture of each solution was heated with a MW reactor equipped with an autosampler (Discovery 2.0, CEM Corporation, USA) in 35 mL vials with a Teflon cap. To ensure homogeneous mixing and prevent sedimentation, all samples were initially agitated vigorously for 2 min inside the microwave unit using a 9 × 15 mm stir bar at the instrument’s maximum stirring speed. Following this prereaction stirring, the MW-assisted reaction commenced, operating at 150 W for 80 min and at a constant temperature of 160 °C. Upon completion of the reaction, compressed air was used to cool the vials to 50 °C. Then, the samples from the vials were transferred to a Petri dish and left in the lab for 3 days to allow for crystal formation and the evaporation of the volatile solvent. Finally, each sample was washed with acetone three times and dried at 50 °C to obtain the hybrid perovskites.

**Table 1:** Chemical Reagents Used in the Synthesis of Hybrid Perovskites

alkylammonium chloride	metal Salt	perovskite (C_n_H_2n+1_NH_3_)_2_MX_4_
C_12_H_25_NH_3_Cl	ZnCl_2_ anhydrous	(C_12_H_25_NH_3_)_2_ZnCl_4_
	CuCl_2_ dihydrate	(C_12_H_25_NH_3_)_2_CuCl_4_
	MnCl_2_ anhydrous	(C_12_H_25_NH_3_)_2_MnCl_4_
2 C_n_H_2n+1_NH_3_X + MX_2_ → (C_n_H_2n+1_NH_3_)_2_MX_4_		

**Figure 1:**
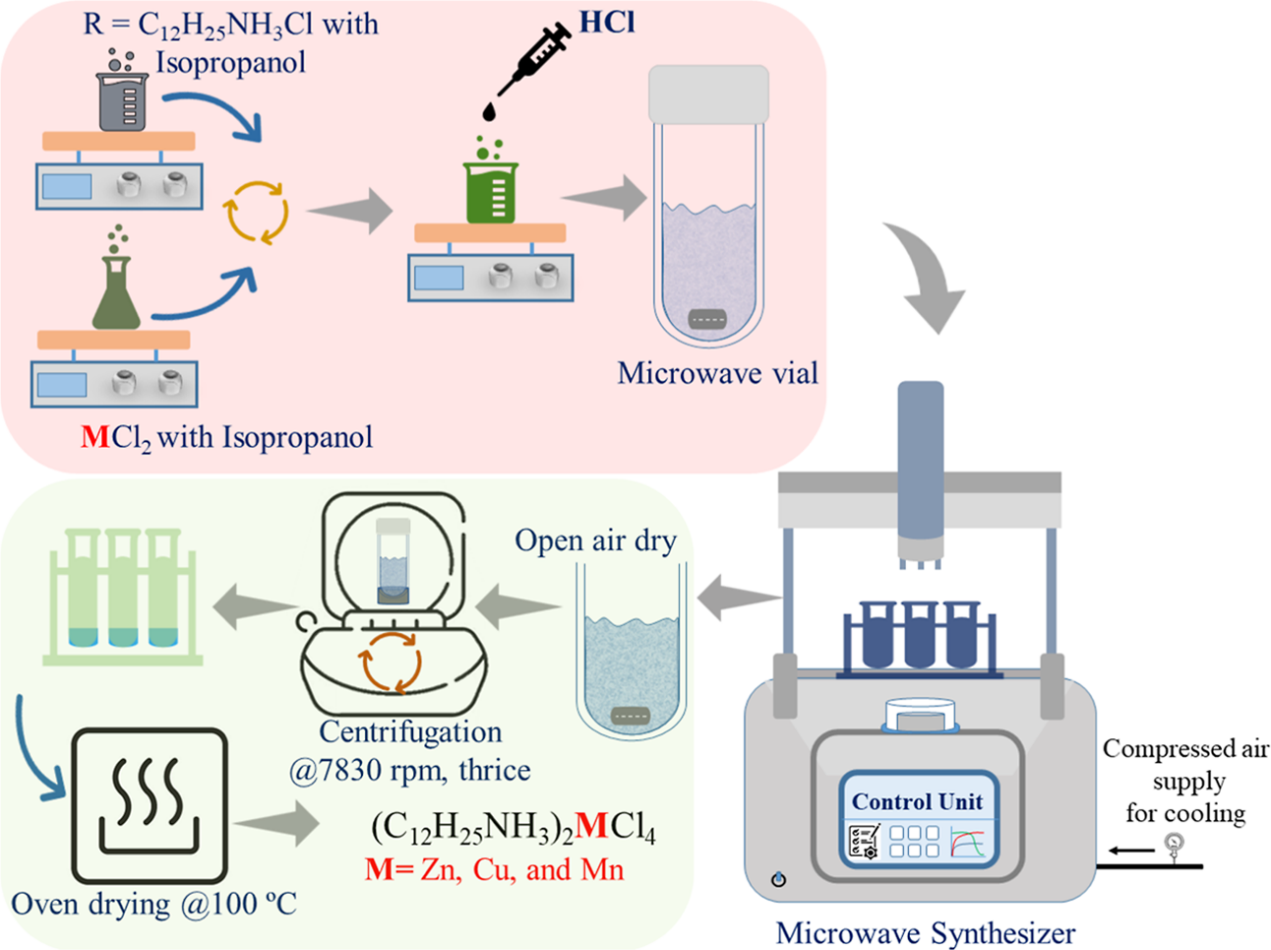
Schematic of the microwave-assisted synthesis of hybrid perovskites (C_12_H_25_NH_3_)_2_MCl_4_ (M = Zn, Cu, and Mn).

### 2.3 Characterization of Hybrid Perovskites

#### 2.3.1 XRD Analysis

X-ray diffraction (XRD) analysis of the MW-assisted hybrid perovskites was performed before and after phase transition using an XRDynamic 500 diffractometer (Anton Paar, Austria) equipped with a Cu Kα_1_ radiation detector (λ = 1.54056 Å). The diffraction patterns were recorded in the 1.5–50° 2θ range with a step size of 0.01°, using an evacuated beam path to reduce background scattering. The XRD measurements were performed in air using the TTK-600 nonambient chamber (Anton Paar, Austria) at 25 °C (pretransition) and 90 °C (post-transition).

#### 2.3.2 FTIR Spectrometer

An INVENIO FTIR spectrometer (Bruker, Germany) was used to obtain the FTIR spectra of the solid hybrid perovskite samples to identify their molecular structures and characterize chemical bonds. All measurements were recorded in transmittance mode, and the spectra were recorded using OPUS software in the wavenumber range of 400–4000 cm^–1^.

#### 2.3.3 SEM Images and EDS Mapping

SEM was employed to examine the surface morphology of the hybrid perovskites. The analysis was performed at ambient temperature using a Hitachi S-4800 (Hitachi High-Tech Corporation, Japan) operated in high-vacuum mode using a secondary electron detector with an acceleration voltage ranging from 0.5 to 30 kV. A piece of copper tape (1 cm^2^) was fixed on an aluminum stub, and approximately 1–2 mg of the sample was deposited on it. Additionally, Energy dispersive X-ray spectroscopy (EDX) equipped with a drift detector was used to obtain EDX spectra and elemental mapping, providing qualitative and semiquantitative elemental information.

#### 2.3.4 DSC Analysis

Differential Scanning Calorimetry (DSC) was conducted to investigate the enthalpy of the solid–solid phase changes of the samples. The measurements were carried out using a DSC25 (TA Instruments, USA). The samples were subjected to a controlled heating program (30 to 180 °C) with a constant heating rate of 5 °C/min under an inert atmosphere of N_2_ during the measurement.

#### 2.3.5 Thermogravimetric Analysis (TGA)

Thermogravimetric analysis (TGA) was performed to evaluate the thermal stability and decomposition behavior of the samples. The measurements were conducted using a TGA/DSC 3+ thermal analysis system (METTLER TOLEDO, Switzerland). The analysis was performed from 40–600 °C at a heating rate of 10 °C/min under a nitrogen flow of 50 mL/min to ensure an inert atmosphere. The mass loss was recorded as a function of temperature, and the corresponding derivative mass loss curve (%/°C) was obtained to identify the temperature ranges and rates of the decomposition processes.

## 3 Results and Discussion

### 3.1 XRD Results

The structural analysis and phase purity of (C_12_H_25_NH_3_)_2_MCl_4_ (M = Zn, Cu, and Mn) were studied using XRD. The diffractograms shown in Figure 2, reveal well-defined crystalline features with characteristic peaks that are consistent with the XRD patterns of hybrid perovskites, specifically the (C_12_H_25_NH_3_)_2_MCl_4_ family (M = Zn, Cu, Mn). The match between the experimental peaks and those reported for analogous compounds supports the successful synthesis of the targeted hybrid perovskite structures. The XRD peaks for (C_12_H_25_NH_3_)_2_ZnCl_4_ showed different major peaks at the 2θ values (*d* spacing) of 2.94° (30.0 Å), 3.93° (22.5 Å), 8.07° (10.9 Å), 12.23° (7.2 Å), 16.40° (5.4 Å), 20.62° (4.3 Å), 24.82° (3.6 Å), 29.10° (3.1 Å), 33.42° (2.7 Å), and 37.78° (2.4 Å). These XRD peaks are in line with previous studies for (C_12_H_25_NH_3_)_2_ZnCl_4_.^42^ For the case of (C_12_H_25_NH_3_)_2_CuCl_4_, the XRD peaks were obtained at 3.04° (29.1 Å), 6.08° (14.5 Å), 9.12° (9.7 Å), 12.19° (7.3 Å), and 15.24° (5.8 Å), which also matches the patterns of previous reports on this material.^22,43^ Finally, for the case of (C_12_H_25_NH_3_)_2_MnCl_4_ the peaks were obtained at 2.91° (31.19 Å), 5.83° (15.33 Å), 8.76° (10.17 Å), 11.70° (7.6 Å), 14.65° (6.0 Å), 20.57° (4.3 Å), 26.56° (3.4 Å), 29.58° (3.0 Å), 32.69° (2.7 Å), 35.67° (2.5 Å), and 38.76° (2.3 Å), in line with the XRD patterns previously reported.^5,44–46^ The presence of well-defined and intense peaks indicates good crystallinity. To further investigate phase transition behavior, XRD patterns were recorded in a nonambient chamber at 90 °C under atmospheric conditions. Following the solid–solid transition, significant structural rearrangements were evidenced by the emergence, disappearance, or shifting of several low- and midangle reflections. These crystallographic changes indicate a reorganization of layer stacking and modifications in crystal symmetry; however, the specific nature of these shifts varied across the samples. While the Cu- and Mn-based hybrids exhibited a shift toward lower angles—indicating an increase in *d*-spacing—the Zn-based sample followed the opposite trend. This divergence in peak shifts is rooted in the fundamental differences in dimensionality and rigidity of the respective inorganic frameworks. In the manganese and copper hybrids, the inorganic component consists of two-dimensional (2D) corner-sharing MCl_6_^2–^ octahedral layers. These continuous sheets act as rigid substrates that restrict the rotational freedom of the units. Consequently, as temperature rises, intensified lattice vibrations cause the unit cell to expand, thereby increasing the interplanar spacing. In contrast, the shift toward higher angles in the zinc-based hybrid indicates lattice contraction.^47^ This is a direct consequence of the unique coordination geometry of Zn^2+^, which forms isolated, zero-dimensional (0D) ZnCl_4_^2–^ tetrahedral units rather than continuous sheets. Because these tetrahedra are not covalently linked, they are “under-constrained” and possess significantly more rotational and vibrational freedom than corner-sharing octahedra. Upon heating, the rocking and liberational motions of these isolated units can effectively hinge the framework, pulling the inorganic layers closer together.^48^ This mechanism is a documented feature of isolated tetrahedral systems, where the thermal activation of specific vibrational modes results in an overall reduction of unit cell dimensions before the phase transition. In summary, the XRD analysis reveals a clear structural divergence across the series: while the Mn- and Cu-based hybrids successfully formed the intended 2D layered perovskite lattices, the Zn-based analogue adopted a 0D structure characterized by isolated tetrahedral ZnCl_4_^2–^ units.

**Figure 2:**
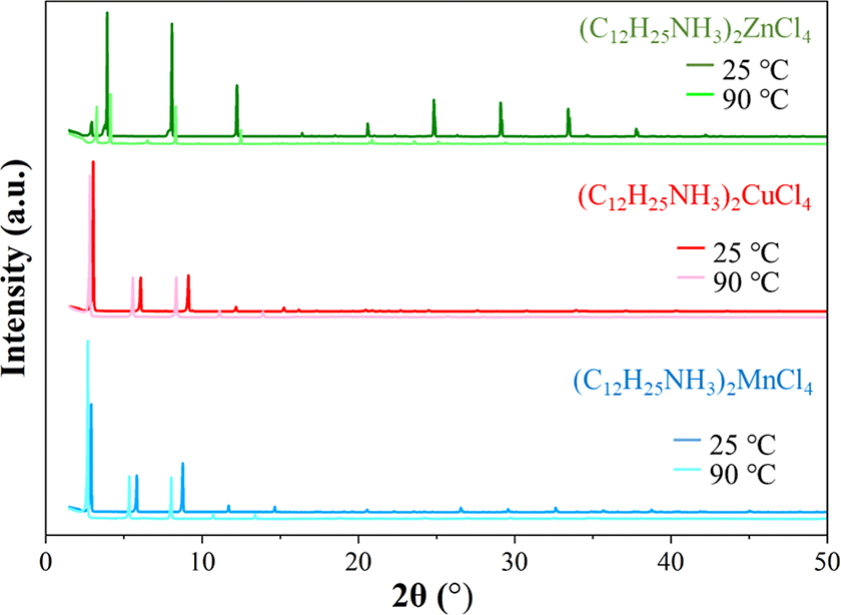
XRD patterns of hybrid perovskites (C_12_H_25_NH_3_)_2_ZnCl_4_, (C_12_H_25_NH_3_)_2_CuCl_4_, and (C_12_H_25_NH_3_)_2_MnCl_4_ below (25 °C) and above (90 °C) the phase transition temperature.

### 3.2 FTIR Results

The FTIR spectra of the Mn-, Cu-, and Zn-based perovskites, shown in Figure 3, reveal a series of characteristic absorption bands that confirm the presence of organic and amine functional groups coordinated with the metal centers. The Mn and Cu based perovskites exhibit distinct rocking vibrations of methylene (R–CH_2_–R) groups at 720 and 728 cm^–1^, respectively, while the Zn based perovskites show these peaks slightly shifted to lower wavenumbers (717 and 724 cm^–1^). The NH_3_^+^ wagging vibrations are clearly observed at 769 cm^–1^ for both Mn and Cu-based perovskites and at 761 cm^–1^ for the Zn-based perovskite. All samples show a characteristic terminal C–C stretching vibration around 887 cm^–1^. All the samples show a peak with a weak intensity band at approximately 1215 cm^–1^ corresponding to C–N stretching, while another weak absorption near 1377 cm^–1^ is attributed to the symmetric bending of methyl (CH_3_) groups. In the range of 1463–1472 cm^–1^, all samples exhibit two distinct peaks assigned to the scissoring and bending vibrations of R–CH_2_–R, reflecting the integrity of the alkyl group.^49,50^ The symmetric deformation of NH_3_^+^ appears at ∼1491 cm^–1^ for Mn and Cu, whereas Zn shows a slightly shifted peak at 1480 cm^–1^. A weak and broad absorption band between 1580 and 1590 cm^–1^ corresponds to the asymmetric deformation of NH_3_^+^, confirming the presence of amine-related vibrations across all samples.^51^ Furthermore, the symmetric and asymmetric stretching vibrations of R–CH_2_–R are identified at 2849 and 2871 cm^–1^, respectively, while those of R–CH_3_ appear at 2917 and 2955 cm^–1^, signifying the organic–inorganic ionic materials.^22,52^ In addition, a distinct difference in the FTIR spectra of these compounds is observed: a clear valley appears at 1740 cm^–1^ for the Zinc and Copper samples, whereas this feature is entirely absent in the Manganese sample. This spectroscopic divergence indicates a fundamental difference in the rigidity of the headgroup anchoring. Based on this observation, it is postulated that the presence of the 1740 cm^–1^ peak in the Zn and Cu lattices allows for a sudden structural collapse during melting.^53^ This collapse is driven by the formation of gauche–gauche defects (large, messy bends that tangle the chains), leading to a sharp transition into total disorder. In contrast, the absence of this peak in the Mn sample suggests a much stiffer environment that prevents such chaos.^53^ Instead, the Mn system undergoes a gradual transition through gauche–trans–gauche kinks (small, single steps that keep the chains mostly straight), allowing the material to maintain a higher degree of structural order during the melting process.^53^ Finally, a broad band observed within the 3400–3500 cm^–1^ region in Zn and Cu based perovskites, and the spectra are attributed to the N–H stretching of primary amines,^51^ indicating the presence of surface-bound or coordinated amine groups, which play a crucial role in stabilizing the metal–organic structure. Moreover, the stretching vibrations associated with the inorganic elements (Zn, Cu, and Mn) with Cl bonds occur in the far-infrared region, which lies outside the FTIR detection range; therefore, these signals do not appear in the spectrum.^28^

**Figure 3:**
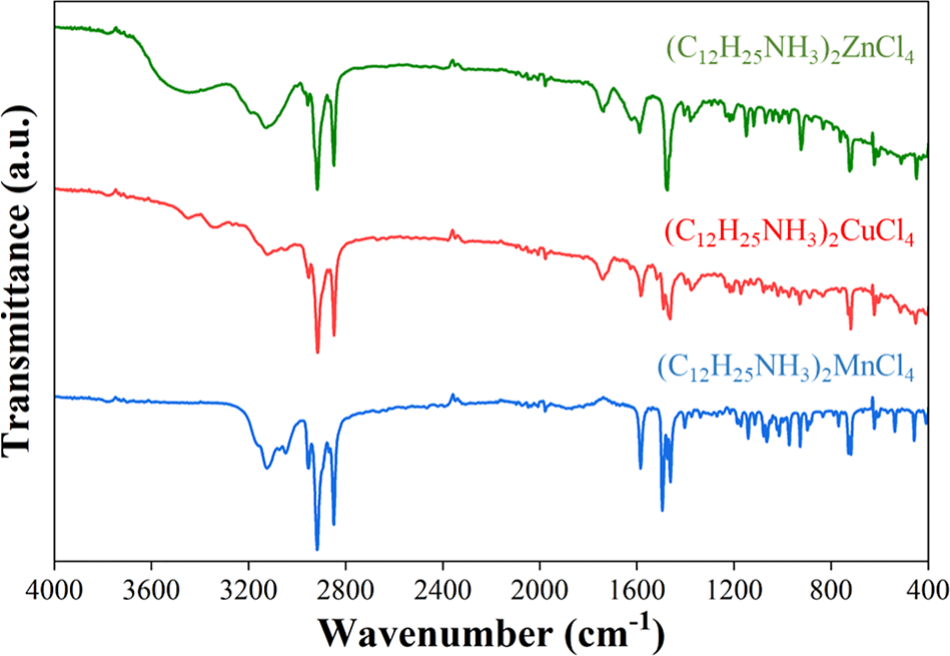
FTIR spectra of hybrid perovskites (C_12_H_25_NH_3_)_2_ZnCl_4_, (C_12_H_25_NH_3_)_2_CuCl_4_, and (C_12_H_25_NH_3_)_2_MnCl_4._.

### 3.3 SEM Images and EDS Mapping

The surface morphology of MW-synthesized perovskites was studied using SEM and is displayed in Figure 4. The obtained results indicate that all samples exhibit a planar, flake-like morphology with a high surface-to-volume ratio, in line with the previous studies.^27,28,54^ The hybrid perovskite particles form flakes with sizes ranging from approximately 1 to 20 μm, exhibiting a nonuniform, polydisperse distribution.

**Figure 4:**
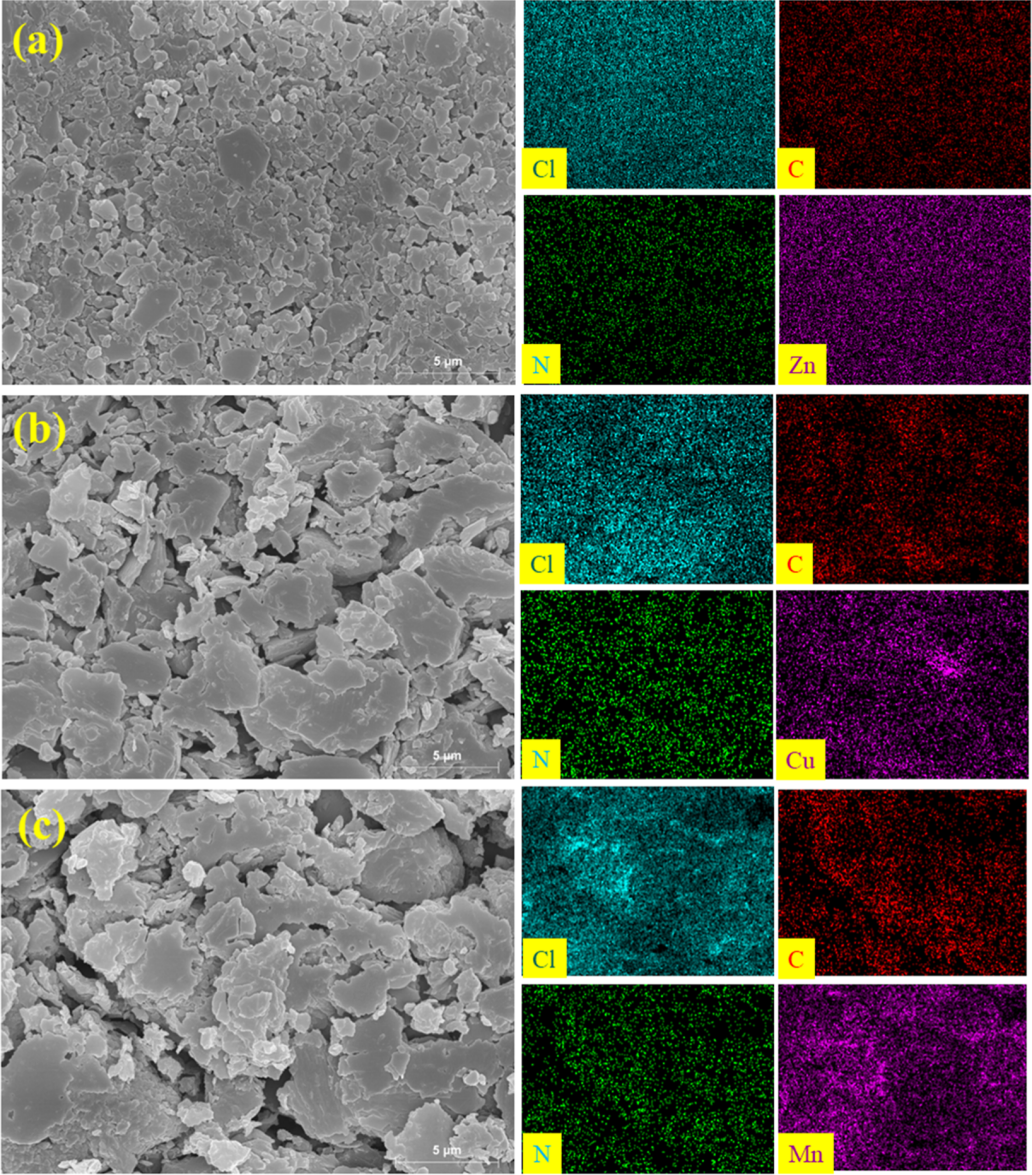
Surface morphology of (a) (C_12_H_25_NH_3_)_2_ZnCl_4_, (b) (C_12_H_25_NH_3_)_2_CuCl_4_, and (c) (C_12_H_25_NH_3_)_2_MnCl_4_ using SEM and EDS mapping.

Energy Dispersive X-ray Spectroscopy (EDX) mapping of the sample was performed for Zn, Cu, and Mn-based perovskite and is shown in Figure 4 (a, b, and c, respectively). EDX confirms the homogeneous distribution of each metal (Zn, Cu, Mn) as well as N, C, and Cl throughout the sample. The EDS spectra (Figure 5) revealed that the Zn-based perovskite contained (atom ±standard deviation) (1.5 ± 0.3)% Zn, (5.6 ± 0.4)% Cl, and (26.2 ± 0.6)% C. In comparison, the Cu-based perovskite contained (1.4 ± 0.2)% Cu, (4.3 ± 0.3)% Cl, and (27.8 ± 0.4)% C, whereas the Mn-based perovskite showed (0.93 ± 0.07)% Mn, (3.48 ± 0.18)% Cl, and (29.8 ± 1.7)% C. These values are in close agreement to the theoretical molecular structure of (C_12_H_25_NH_3_)_2_MCl_4_ - 1.15% M, 27.58% C, and 4.59% Cl, determined by dividing the number of atoms of each element (M: 1, C: 24 and Cl: 4) by the total number of atoms in the formula (87), given that EDS is a semiquantitative technique with limited accuracy for light elements. No other elements were observed in EDS spectra, suggesting the purity of the hybrid perovskite samples. Overall, the combination of SEM, EDS, FTIR, and XRD results provides direct evidence of well-formed hybrid perovskites of crystalline structures with uniform elemental composition, and validates the successful synthesis via microwave-assisted method of the intended organic–inorganic hybrid compounds.

**Figure 5:**
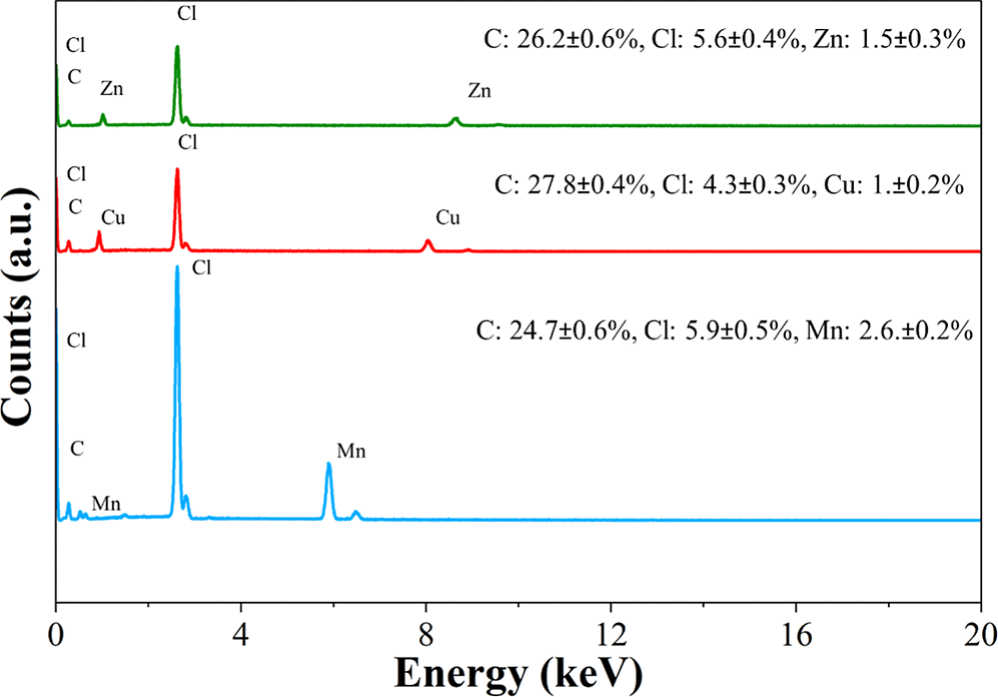
EDS spectra of hybrid perovskites (C_12_H_25_NH_3_)_2_ZnCl_4_, (C_12_H_25_NH_3_)_2_CuCl_4_, and (C_12_H_25_NH_3_)_2_MnCl_4_.

### 3.4 DSC Results

The DSC analysis of the synthesized samples is displayed in Figure 6, showing different endothermic and exothermic peaks corresponding to the solid–solid phase transitions of the hybrid perovskites.^29,44^ These results confirm that all three samples synthesized through the MW method showed a reversible solid–solid phase transition with a controlled order–disorder mechanism of the long-chain alkylammonium bilayers reported in earlier studies.^24,55^ The enthalpy of each perovskite was measured over a temperature range of 30 °C during both heating and cooling, spanning from just before the onset of phase transition to after its completion. The results are reported in Table 2 for improved comparability. It should be noted that although minor additional peaks may be observed in some DSC curves, only the principal reversible solid–solid transition with the highest enthalpy was analyzed, as the other thermal events correspond to weak secondary structural rearrangements with negligible contribution to the overall energy storage performance.

**Figure 6:**
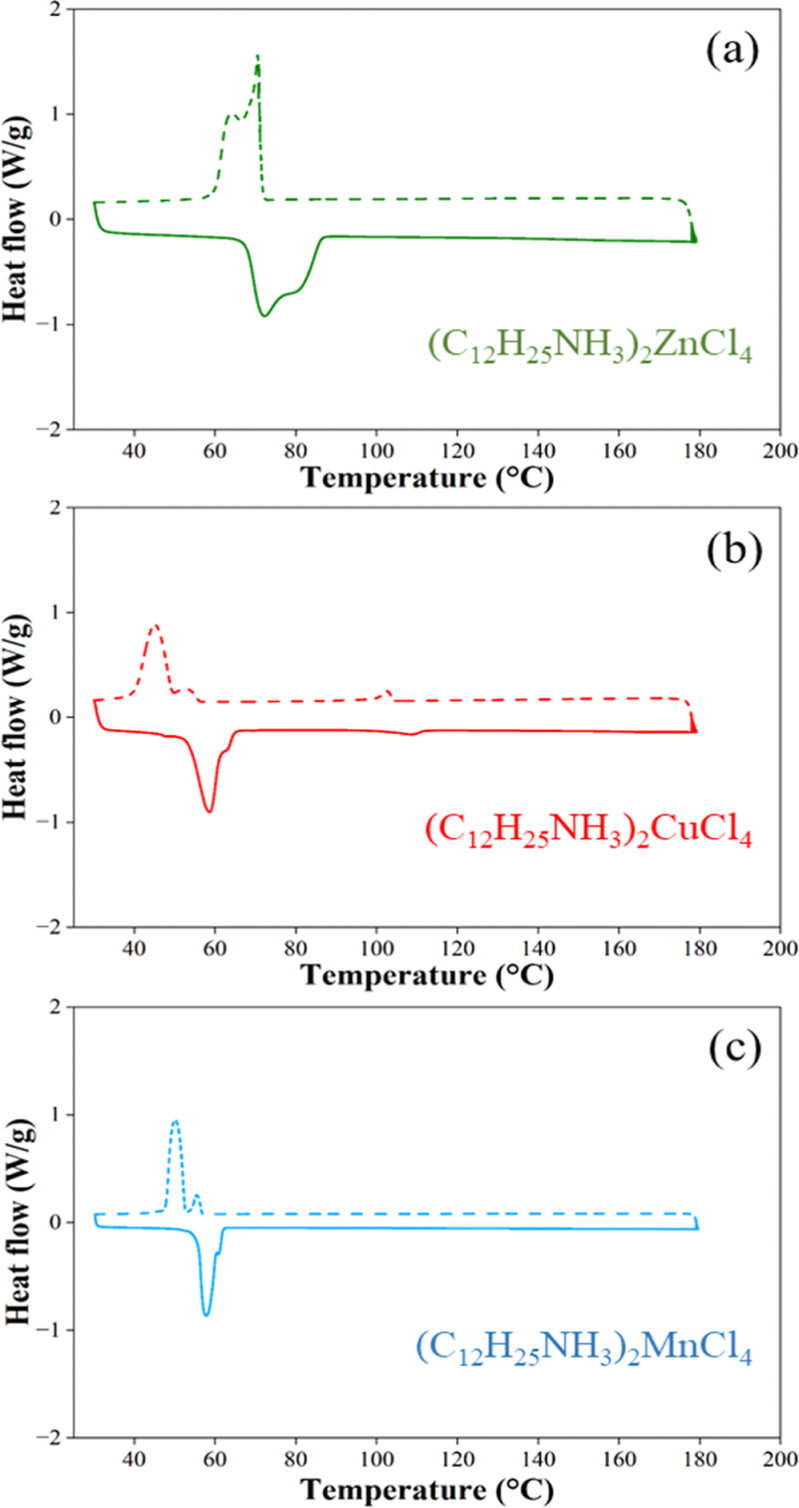
DSC thermograms showing solid–solid phase transitions of hybrid perovskites: (a) (C_12_H_25_NH_3_)_2_ZnCl_4_, (b) (C_12_H_25_NH_3_)_2_CuCl_4_, and (c) (C_12_H_25_NH_3_)_2_MnCl_4_.

**Table 2:** Properties of the solid–solid Phase Change in Hybrid Perovskites (Heating and Cooling)

sample	ΔH-heating^a^ (J/g)	peak temperature-heating (degC)	ΔH-cooling^a^ (J/g)	peak temperature-cooling (degC)
(C_12_H_25_NH_3_)_2_ZnCl_4_	109.40 ± 1.46	72.39 ± 0.46	112.62 ± 0.23	73.84 ± 0.23
(C_12_H_25_NH_3_)_2_CuCl_4_	58.62 ± 0.1	57.90 ± 0.26	60.14 ± 0.88	46.82 ± 0.26
(C_12_H_25_NH_3_)_2_MnCl_4_	97.86 ± 0.55	57.97 ± 0.07	99.28 ± 0.10	51.37 ± 0.17

a Both enthalpies of solid–solid phase transitions in the heating and cooling stages are measured over a temperature range of 30 °C, over the phase transition range.

During heating, (C_12_H_25_NH_3_)_2_ZnCl_4_ exhibited a transition peaking at 72.4 °C with a latent heat uptake of 109.4 J/g, while the reverse cooling cycle displayed an exothermic peak at 73.84 °C, corresponding to the release of 112.62 J/g of stored thermal energy (Figure 6a). In the case of (C_12_H_25_NH_3_)_2_CuCl_4,_ the solid–solid phase transition during heating was observed at a lower temperature of 57.9 °C, with an enthalpy of 58.62 J/g, while the reverse cooling peaks at 46.82 °C with an enthalpy of 60.14 J/g (Figure 6b). Compared to their Zn-based counterparts, Cu-based perovskites exhibit lower phase transition temperatures while maintaining substantial enthalpy, rendering them highly suitable for medium-temperature thermal management. Similarly, (C_12_H_25_NH_3_)_2_MnCl_4_ exhibits a phase transition at ∼58.0 °C during heating (Figure 6c), but showed ∼67% higher enthalpy (97.86 J/g) compared to the (C_12_H_25_NH_3_)_2_CuCl_4_. During the cooling, the phase transition for Mn-based perovskites showed a phase change at ∼51.4 °C with an enthalpy of 99.28 J/g. The temperature discrepancy between heating and cooling cycles is attributed to thermal hysteresis, characteristic of first-order solid–solid transitions. During heating, energy is required to disorder the alkylammonium bilayers, while kinetic delays in chain reorganization shift the transition lower upon cooling. This behavior reflects the intrinsic energy barrier of structural rearrangement and confirms the reversible nature of the transition.^56^

Transition enthalpies generally increase with alkyl chain length up to 12 carbons, but stabilize thereafter.^22,57,58^ In this study, where chain length is constant, thermal variations are driven by inorganic coordination. The Zn-based hybrid exhibits the highest transition temperature and enthalpy (73.1 °C, 111 J/g) because it adopts a distinct 0D tetrahedral structure, unlike the 2D layered octahedral frameworks of Mn and Cu.^19,20,59^ Among the layered systems, the Cu hybrid shows lower values than Mn due to Jahn–Teller distortions, which increase framework flexibility and reduce stability compared to the more symmetric Mn lattice.^20,60^

### 3.5 TGA Results

The thermal stability of the synthesized hybrid perovskites was evaluated via TGA and DTG (Figure 7), revealing a characteristic three-stage mass-loss profile. Thermal stability, quantified by the 5% mass-loss temperature (*T*_d_), followed the trend Zn > Cu > Mn. The Zn-based perovskite exhibited the highest stability (*T*_d_ ∼ 335 °C), due to a different crystalline structure, 0D instead of 2D. In contrast, the Cu-based analogue showed intermediate stability (*T*_d_ ∼ 305 °C), where inherent Jahn–Teller distortions may slightly destabilize the lattice symmetry. The Mn-based compound was the most sensitive, with a significantly lower onset (*T*_d_ ∼ 270 °C) reflecting weaker Mn–Cl coordination and less robust organic–inorganic interactions. DTG curves identified three kinetic stages: an initial shoulder peak (Stage I), a primary decomposition peak (Stage II), and a final structural resolution (Stage III). For the Zn, Cu, and Mn variants, these main Stage II peaks occurred at 400, 370, and 340 °C, respectively. The final residual masses (∼22–27%) correspond to the metal chloride products remaining after the complete combustion of the C_12_H_25_NH_3_ organic chains.

**Figure 7:**
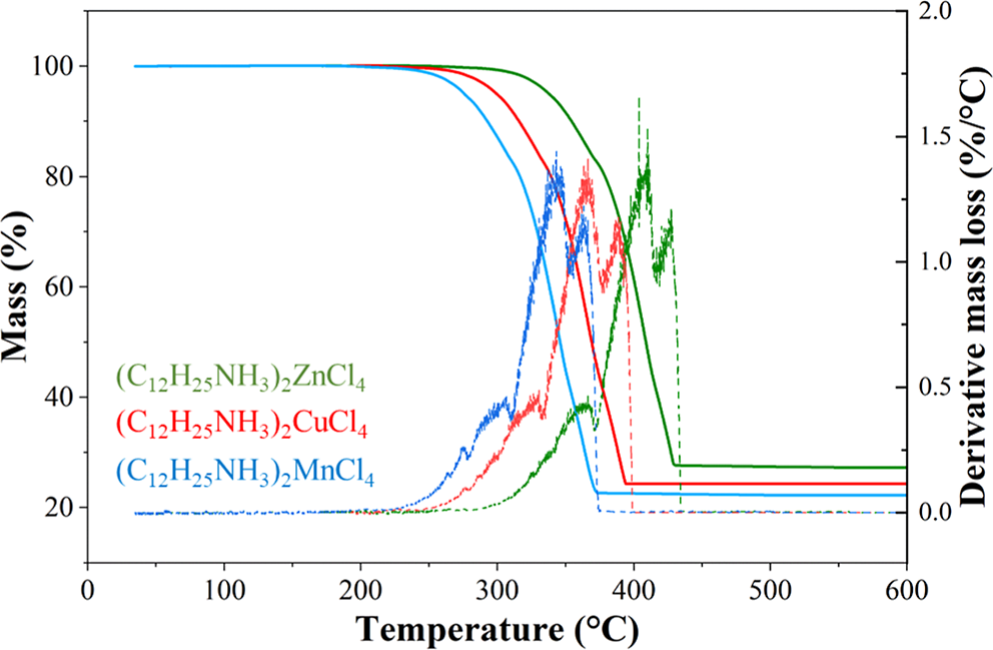
TGA (solid lines) and derivative mass loss (dashed lines) curves for hybrid perovskites: (C_12_H_25_NH_3_)_2_ZnCl_4_, (C_12_H_25_NH_3_)_2_CuCl_4_, and (C_12_H_25_NH_3_)_2_MnCl_4_.

## 4 Conclusion

In this study, we developed a novel microwave-assisted synthesis route for hybrid perovskites ((C_12_H_25_NH_3_)_2_MCl_4_, where M = Zn, Cu, Mn) to evaluate their feasibility as solid–solid phase change materials (PCMs). X-ray diffraction (XRD) confirmed high-purity crystalline structures for all samples, while FTIR analysis verified a chemical organization consistent with traditional synthesis methods. Surface morphology and elemental mapping further corroborated the successful formation of the hybrid frameworks. Notably, while the synthesis was intended to produce a homologous series of layered structures, the Zn-based compound diverged into a distinct tetrahedral geometry. Despite this structural variation, the Zn-based perovskite exhibited the highest latent heat (∼110.0 J/g) with an advantageous phase change temperature of 73.0 °C, confirming its high potential for TES applications. In comparison, the Mn-based and Cu-based analogues demonstrated enthalpies of ∼98 J/g (at 51–59 °C) and ∼59 J/g (at 46.8–57.9 °C), respectively. Furthermore, TGA results indicated robust thermal stability, with decomposition onsets at 335 °C (Zn), 305 °C (Cu), and 225 °C (Mn). These variations stem from the central metal ion’s influence on metal–chloride bond strength and the resulting inorganic architecture. Specifically, the structural differences—ranging from the tetrahedral ZnCl_4_ geometry to the octahedral MnCl_6_ framework and the Jahn–Teller distorted Cu-based lattice—directly dictate the material’s thermal behavior. The ability to tune phase transition properties and stability through compositional and structural control makes these perovskites ideal for medium-temperature thermal energy storage. Additionally, the adoption of microwave synthesis provides significant economic and scalability advantages, including reduced reaction times and superior heating uniformity. This solvent-efficient, modular approach addresses key barriers to commercialization, positioning these tunable PCMs as high-performance, industrially viable solutions for thermal regulation systems.
